# IPO7 promotes pancreatic cancer progression via regulating ERBB pathway

**DOI:** 10.1016/j.clinsp.2022.100044

**Published:** 2022-05-16

**Authors:** Ming Li, Dongqiang Xu, Yijun Zhan, Shiyun Tan

**Affiliations:** a Department of Gastroenterology, Renmin Hospital of Wuhan University, Hubei Province, China; b Department of Gastroenterology, Xiangyang Central Hospital, Affiliated Hospital of Hubei University of Arts and Science, Hubei Province, China

**Keywords:** Pancreatic cancer, IPO7, ERBB pathway

## Abstract

**Background:**

Importin 7 (IPO7) belongs to the Importin β family and is implicated in the progression of diverse human malignancies. This work is performed to probe the role of IPO7 in pancreatic cancer development and its potential downstream mechanisms.

**Methods:**

IPO7 expression in PC and paracancerous tissues were measured using Immunohistochemistry (IHC) staining and qRT-PCR. Western blotting was utilized to detect the expression level of IPO7 in PC cells and immortalize the pancreatic ductal epithelial cell line. After constructing the IPO7 overexpression and knockdown models, the effect of IPO7 on the proliferation of PC cells was analyzed by the CCK-8 and EdU assay. The migration and invasion of PC cells were examined by wound healing assay and Transwell experiment. The apoptosis rate of PC cells was analyzed by flow cytometry and TUNEL assay. The Gene Set Enrichment Analysis (GSEA) was used to determine the enrichment pathways of IPO7. The effect of IPO7 on the ERBB2 expression was determined using Western blotting. A xenograft mouse model was applied to investigate the carcinogenic effect of IPO7 *in vivo*.

**Results:**

IPO7 expression was remarkably elevated in the cancer tissues of PC patients. IPO7 overexpression remarkably enhanced PC cell proliferation, migration and invasion and suppressed apoptosis, while knockdown of IPO7 exerted the opposite effect. Mechanistically, IPO7 facilitated the malignant phenotype of PC cells by up-regulating ERBB2 expression. In addition, knockdown of IPO7 inhibited tumor growth and lung metastasis *in vivo.*

**Conclusion:**

IPO7 can act as an oncogenic factor and accelerate PC progression by modulating the ERBB pathway.

## Highlights

• IPO7 remarkably enhanced pancreatic cancer cell proliferation, migration and invasion and suppressed apoptosis.
• IPO7 facilitated the malignant **phenotype** of pancreatic cancer cells by up-regulating ERBB2.
• Knockdown of IPO7 inhibited tumor growth and lung metastasis *in vivo*.

## Introduction

Pancreatic Cancer (PC) is a lethal type of cancer with a high mortality rate.^1^^,^^2^ Reportedly, over 95% of PC patients have distant metastasis.^3^ The 5-year survival rate of PC patients is only 2%-9%.^1^^,^^2^ Therefore, further investigation of the mechanisms of PC progression is essential to improve the prognosis of PC patients.

Importin-7 (IPO7) belongs to the importin-β family. Importin β family members are nucleoplasmic transport receptor proteins widely found in eukaryotes.^4^ Importin β family members can induce nucleoplasmic translocation by binding with the substrate or junction proteins at the C-terminus, RanGTP at the N-terminus, and nuclear pore proteins at the intermediate site.^5^^,^^6^ Importin β family members belong to a conserved family of proteins with similar molecular weight and multiple HEAT-repeat domains.^7^^,^^8^ Reportedly, IPO7 is carcinogenic in several human malignancies, such as colorectal cancer and glioblastoma.^9^^,^^10^ In the present study, bioinformatics implies that IPO7 expression is up-modulated in PC tissues, and it is correlated with poor prognosis of PC patients. Nonetheless, the role of IPO7 in PC progression and its mechanism are blurred.

ERBB family, known as a class of epidermal growth factor receptors, belongs to the tyrosine kinase receptors and includes ERBB1, ERBB2, ERBB3 and ERBB4.^11^ Overexpression of some members of the ERBB family, especially ERBB2 (Her2), is strongly associated with high aggressiveness and poor prognosis of human malignancies.^12^ ERBB2 exerts a crucial regulatory role in the development of most tumors.^13^^,^^14^ Importantly, ERBB2 is also a crucial oncogene to facilitate the progression of PC.^15^ Nonetheless, the mechanism of ERBB2 dysregulation in PC is undefined.

In this work, the biological functions of IPO7 in regulating the malignant phenotype of PC cells were probed, and IPO7 was revealed to be remarkably up-modulated in PC tissues and cells. It was also revealed that IPO7 markedly enhanced the growth, migration, and invasion of PC cells and repressed their apoptosis. Additionally, IPO7 could positively modulate the ERBB2, and knockdown of IPO7 inhibited tumor growth and lung metastasis *in vivo*.

## Materials and methods

### Collection of human tissue samples

PC tissues and paired para-cancerous tissues were obtained from 60 PC patients during surgical resection. The patients participating in this research signed written informed consent before surgery. None of the patients had received radiotherapy and chemotherapy prior to surgery, and all PC tissues were confirmed by pathologists. This research was endorsed by the Ethics Committee of Renmin Hospital of Wuhan University. After collecting tissue samples, all of the samples were stored in liquid nitrogen for rapid freezing and preserved at -80°C.

### Cell culture and transfection

Immortalized pancreatic ductal epithelial cell line (hTERT-HPNE) and four human PC cell lines (PANC-1, HPAC, BxPC-3, and Capan-2) were procured from American Type Culture Collection (ATCC, Rockville, MD, USA). All of the cells were cultured in Roswell Park Memorial Institute (RPMI)-1640 medium containing 10% Fetal Bovine Serum (FBS) (Gibco; Thermo Fisher Scientific, Inc., Waltham, MA, USA) at 37°C, 5% CO_2_. The cells in the logarithmic growth phase were transferred into a 6-well plate at 6 × 10^5^/well, and pcDNA3.1 empty vector (pcDNA), pcDNA3.1-IPO7 (pcDNA-IPO7), negative control siRNA (NC-siRNA) and two IPO7 siRNAs (IPO7-siRNA#1 and IPO7-siRNA#2) (GenePharma Co., Ltd, Shanghai, China) were transfected into cells. After 48h, the cells were collected, and quantitative Real-Time Polymerase Chain Reaction (qRT-PCR) was executed to validate the transfection efficiency.

### qRT-PCR

TRIzol reagent (Invitrogen, Carlsbad, CA, USA) was utilized to extract total RNA from PC tissues or cells. A High-Capacity cDNA Reverse Transcription kit (Thermo Fisher Scientific, Inc., Waltham, MA, USA) was adopted for first-strand cDNA synthesis. β-actin was used as an internal reference and qRT-PCR was performed using a SYBR Premix Ex Taq™ Kit (Takara Biotechnology Co. Ltd., Dalian, China) on an Applied Biosystems 7500 assay system (Applied Biosystems Ltd., Waltham, MA, USA) for qRT-PCR, and the relative expression was calculated by the 2^−ΔΔCT^ method. The primer sequences were shown in Table 1.

**Table 1:** The primer sequences for qRT-PCR.

Gene	Sequences	
ERBB2	Forward	5′-TGCAGGGAAACCTGGAACTC-3′
	Reverse	5′-ACAGGGGTGGTATTGTTCAGC-3′
IPO7	Forward	5′-CCCCAACACCATTATCGAGGC-3′
	Reverse	5′-AGAGACTTGTGTGCTTCATTGAG-3′
β-actin	Forward	5′-CTACGTCGCCCTGGACTTCGAGC-3′
	Reverse	5′-GATGGAGCCGCCGATCCACACGG-3′

### Immunohistochemistry (IHC)

Paraffin-embedded PC tissues were cut into 4-μm-thick sections. Subsequently, they were dewaxed in xylene, rehydrated by graded concentrations of alcohol, and heated in citrate buffer (pH 6.0) for 30 min. Endogenous peroxidase activity was blocked by treatment with 3% H_2_O_2_ for 10 min, and the sections were then incubated with a blocking solution for 2h. Subsequently, the specimens were incubated overnight with a primary anti-IPO7 antibody (1:100, ab99273, Abcam, Shanghai, China). On the next day, the sections were incubated with secondary antibody (Beyotime, Shanghai, China) for 1h at room temperature. Subsequently, the sections were washed with double distilled water and then stained with 3,3-diaminobenzidine hydrochloride for 1 min. Then the sections were washed with double distilled water again, and the sections were subsequently stained with hematoxylin for 1 min. Finally, the sections were observed under a light microscope.

### Western blotting

The transfected PC cells were lysed in RIPA lysis buffer (Beyotime, Shanghai, China), and the protein concentrations were determined using a BCA Protein Assay Kit (Thermo Scientific, MA, USA). The total protein was separated by SDS-PAGE and transferred to a PVDF membrane (Millipore, Eschborn, Germany). The membranes were then blocked with 5% skimmed milk for 1h. Next, the PVDF membranes were incubated with the primary antibodies anti-IPO7 (1:1000, ab99273, Abcam, Shanghai, China) and anti-ERBB2(1:1000, ab134182, Abcam, Shanghai, China) overnight at 4°C. Subsequently, the membranes were washed by tris buffered saline tween (TBST) and then incubated with secondary antibodies (1:5000, Beyotime, Shanghai, China) for 1h at room temperature. Finally, the PVDF membrane was washed by Tris Buffered Saline Tween (TBST) again, and the protein bands were developed by the ECL chemiluminescence kit (Millipore, Eschborn, Germany).

### Cell counting kit-8 (CCK-8) assay

The transfected PC cells were inoculated at 5 × 10^3^ cells/well in a 96-well plate and cultured overnight. 10 μL of CCK-8 reagent (Dojindo Molecular Technologies, Inc., Kyushu, Japan) was supplemented in each well and incubated for 1h at 37°C. Then the absorbance values (optical density at 450 nm wavelength) of the cells in each well were measured on a microplate reader. With this method, the absorbance values of the cells in each well were evaluated at the 24^th^, 48^th^, 72^th^ and 96^th^h, respectively.

### 5-Ethynyl-2-Deoxyuridine (EdU) assay

The transfected PC cells were incubated with 10 μM EdU reagent for 2h in the darkness according to the instruction of an EdU assay kit (Beyotime, Shanghai, China). The cells were then fixed with 4% paraformaldehyde for 20 min; incubated with Phosphate Buffer Saline (PBS) containing 5% bull serum albumin; and labeled with Alexa Fluor 555 for EdU, and Hoechst 33342 for staining the nuclei. After the cells were washed by PBS, the cells were observed using a fluorescence microscope. Proliferation rate (%) = the number of the red fluorescent cells / the number of the blue fluorescent cells  ×  100%.

### Wound healing assay

The transfected PC cells were seeded in 6-well plates at 5 × 10^5^ cells/well, and cultured. When the cells reached about 90% confluence, a linear wound was created by using a 200 μL pipette tip, and then the cells were cultured for 48h in a serum-free medium. At 0 and 48^th^, the wound width at the same position was recorded under the microscope.

### Transwell assay

In the invasion experiment, a layer of Matrigel (Corning, Beijing, China) was used to cover the membrane of Transwell chambers. The transfected PC cells were re-suspended in a serum-free medium and then inoculated into the upper compartment of the Transwell system. RPMI-1640 medium containing 10% FBS was supplemented to the lower compartment. After incubating for 48h, the invasive cells, which were on the below surface of the membrane, were fixed with 4% paraformaldehyde and stained with 0.5% crystal violet solution. The migration experiments were performed as described above, but the membrane was not pre-coated with Matrigel. The number of migrating and invading cells was observed under a Leica DM4000B microscope (Leica, Wetzlar, Germany).

### Flow cytometry

Annexin V-FITC/PI Double Staining Kit (Yeasen Biotech Co., Ltd., Shanghai, China) was used to detect the apoptosis of the transfected PC cells. The PC cells were inoculated in a 6-well plate at 5 × 10^5^ cells/well and cultured overnight, then trypsinized with EDTA-free trypsin, and the cells were collected. Then the cells were rinsed twice with pre-cooled PBS and re-suspended with 100 μL of 1  ×  binding buffer. Then the cells were incubated with 5 μL of Annexin V-FITC staining solution and 10 μL of Propidium Iodide (PI) staining solution for 10 min in the dark. Next, 400 μL of 1  ×  binding buffer was supplemented, and the cells were analyzed by a flow cytometer (BD Biosciences, San Jose, CA, USA).

### Terminal deoxynucleotidyl transferase dUTP nick end labeling (TUNEL) assay

The apoptosis of the transfected PC cells was measured by using the one-step TUNEL apoptosis assay kit (Beyotime, Shanghai, China). Briefly, the transfected cells were fixed with methanol and incubated with 50 μL of TUNEL solution at 37°C for 60 min. Subsequently, the cells were washed with PBS, then stained with DAPI staining solution at 37°C for 15 min, and then washed with PBS again and finally, the cells were observed and counted under a fluorescence microscope.

### Animal experiment

Ten 6-week-old female BALB/c nude mice were applied to establish xenograft tumor models, and approximately 1 × 10^7^ PANC-1 cells were injected subcutaneously into each nude mouse (5 in the control group and 5 in the IPO7 knockdown group). The volume of the tumor was measured every 7 days using digital calipers. The tumor volume was calculated using the formula: 0.5 ×  Length  ×  Height  ×  Width. Then, the mice were sacrificed after 35 days, and the weight of the tumors was measured. Another ten mice were injected with PC cells through the caudal vein (5 in the control group and 5 in the IPO7 knockdown group). After 6 weeks, the nude mice were sacrificed. The lung tissues of mice were removed and fixed with 4% paraformaldehyde and sectioned after paraffin embedding. Subsequently, Hematoxylin-Eosin (HE) staining was performed, and the metastatic nodules were observed and evaluated under the microscope.

### Statistical analysis

SPSS 23.0 (SPSS Inc., Chicago, IL, USA) was employed for the statistical analysis of all data. The results are presented as the mean ± Standard Deviation (SD). Graphing was conducted using GraphPad Prism 8 (GraphPad Prism, Inc., La Jolla, CA, USA). All the data were represented as the mean ± standard deviation. Student's *t*-test and one-way ANOVA were adopted for the statistical analyses; p < 0.05 signified statistical significance.

## Results

### IPO7 expression is up-regulated in PC

**Fig. 1:**
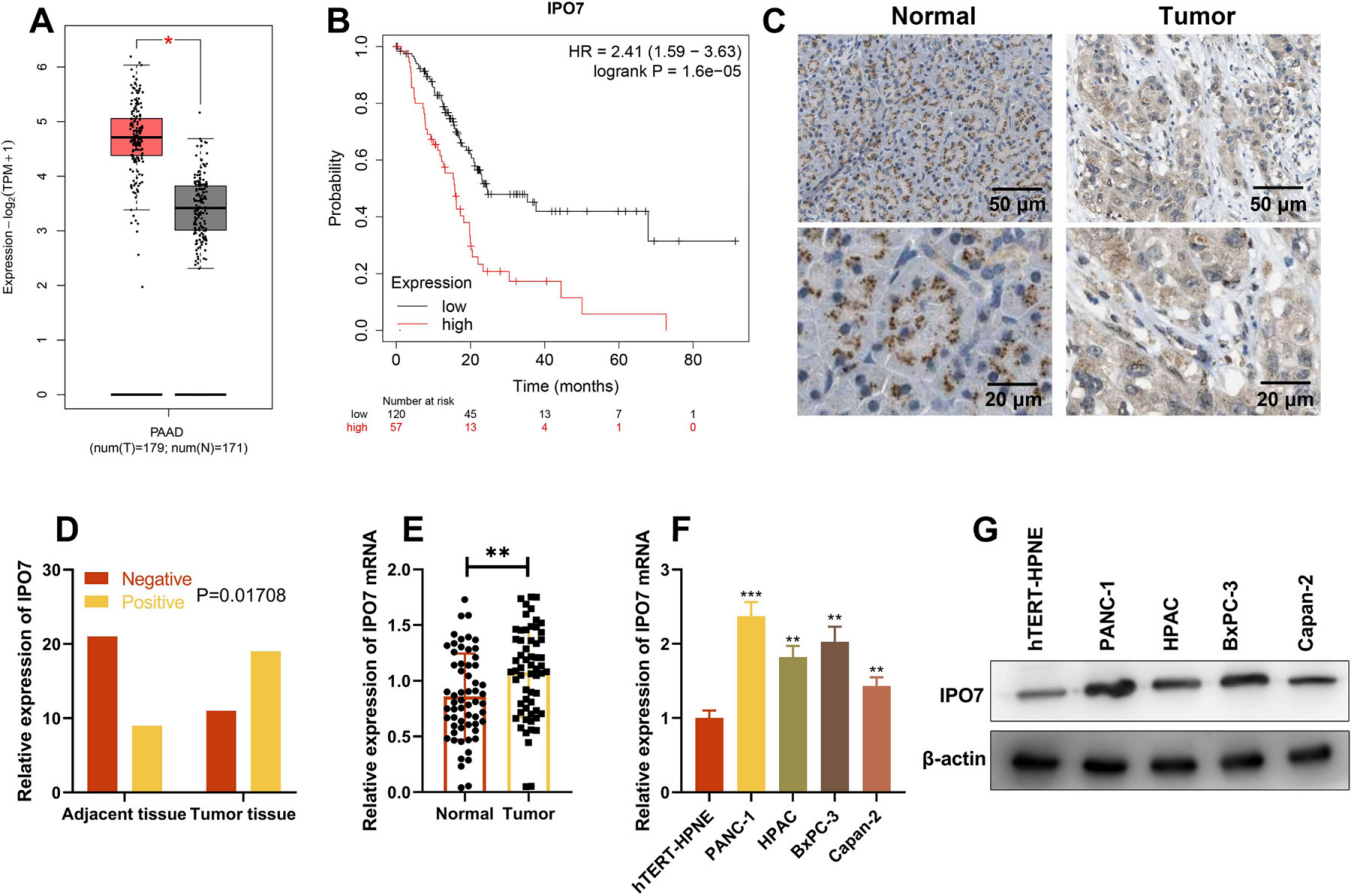
IPO7 expression in PC tissues and cell lines. (A‒B) IPO7 expression was up-regulated in PC tissues and the expression of IPO7 was correlated with the prognosis of PC patients by searching the Gene Expression Analysis Interactive Analysis (GEIPA) database. (C‒D). Immunohistochemistry showed that IPO7 was highly expressed in PC tissues. (E) qRT-PCR showed that IPO7 mRNA expression was up-regulated in PC tissues. F-G. qRT-PCR and Western blotting showed that IPO7 expression was up-regulated in 4 PC cell lines (PANC-1, HPAC, BxPC-3 and Capan-2) compared with the normal pancreatic ductal epithelial cell line (hTERT-HPNE). All of the experiments were performed in triplicate (**p < 0.01; ***p < 0.001).

Gene Expression Profiling Interactive Analysis (GEPIA) database showed that IPO7 expression in PC tissues was remarkably higher than that in normal tissues, and higher expression of IPO7 was correlated with the poor prognosis of PC patients (Fig. 1 A‒B). Then immunohistochemical staining was used to detect the expression of IPO7 protein in the tumor tissue and paracancerous tissue of PC patients, and the representative images are shown in Fig. 1C. The statistical analysis showed that IPO7 expression was remarkably up-regulated in PC tissues compared with that in paracancerous tissues, and the difference was statistically significant (Fig. 1D). Moreover, qRT-PCR was also utilized to detect and analyze IPO7 expression in 60 PC and their corresponding paracancerous tissues, and consistently, the data showed that IPO7 was markedly up-modulated in PC tissues (Fig. 1E). Additionally, IPO7 was revealed to be remarkably higher in PC cell lines (PANC-1, HPAC, BxPC-3, and Capan-2) than in immortalized normal pancreatic ductal epithelial cell line (hTERT-HPNE) by qRT-PCR and Western blotting (Fig. 1F‒G). Based on the median expression of IPO7 mRNA, the enrolled 60 PC patients were classified into two groups (high expression group and low expression group). The results were shown in Table 2, which showed that the high expression of IPO7 was correlated with lymph node metastasis and advanced TNM staging of the patients.

**Table 2:** Relationship between IPO7 expression and clinicopathological characteristics of PC patients.

Parameters	Number	IPO7 expression		p
		High (n = 30)	Low (n = 30)	
Age (years)				
≥ 60	32	14	18	0.300
< 60	28	16	12	
Gender				
Male	34	19	15	0.294
Female	26	11	15	
Clinical stage				
Ⅲ‒Ⅳ	32	21	11	0.009^a^
Ⅰ‒Ⅱ	28	9	19	
Lymphatic metastasis				
Yes	28	18	10	0.038^a^
No	32	12	20	

a p < 0.05.

### IPO7 facilitates PC cell proliferation and restrains cell apoptosis

**Fig. 2:**
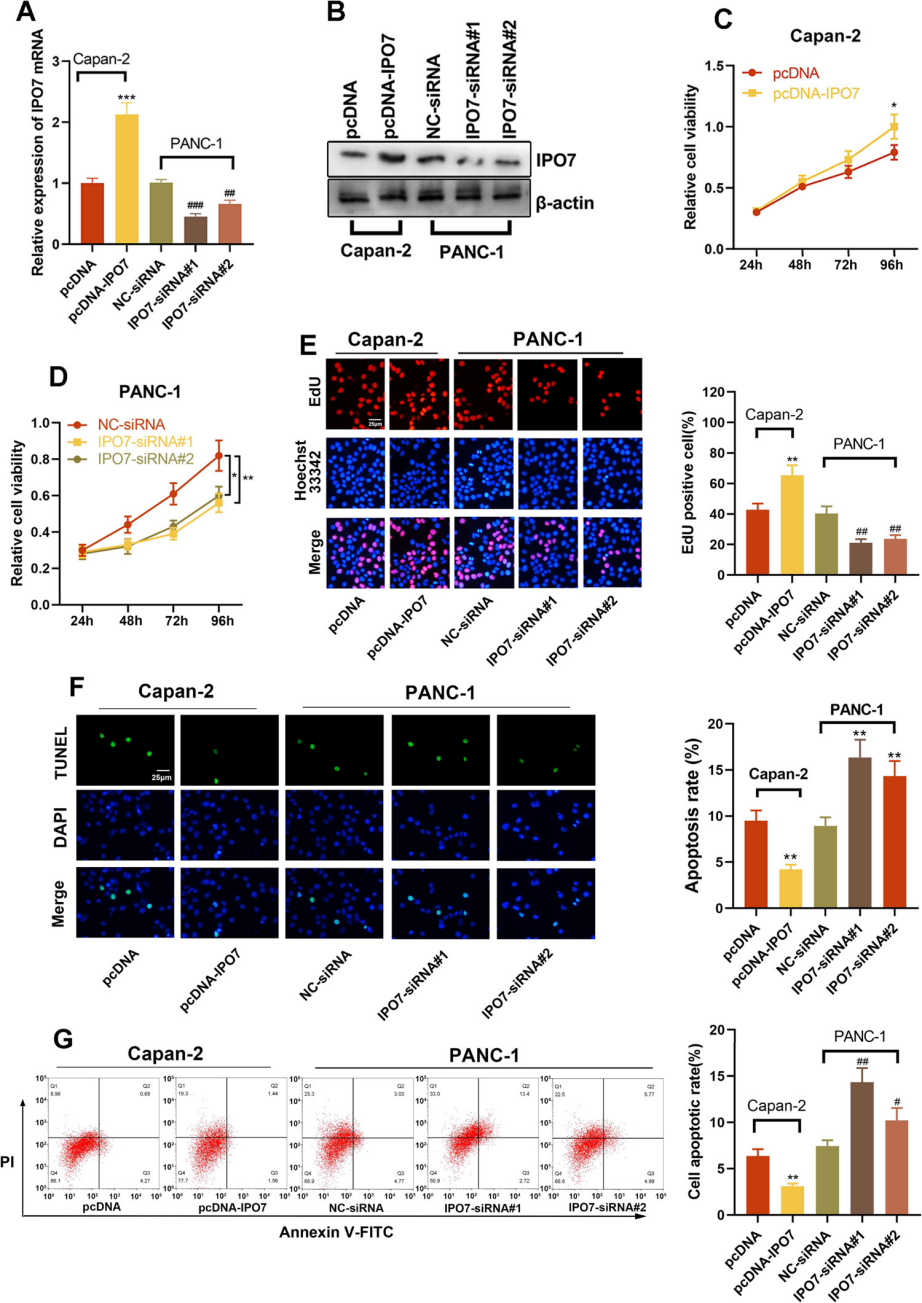
Effect of IPO7 on the proliferation and apoptosis of PC cells. (A‒B) qRT-PCR and Western blotting were adopted to verify the transfection efficiency of pcDNA-IPO7 and IPO7-siRNAs. (C‒E) CCK-8 assay and EdU experiment showed that PC cell proliferation was enhanced in pcDNA-IPO7 group and decreased in the IPO7-siRNA#1 and IPO7-siRNA#2 group. (F‒G) Flow cytometry and TUNEL assay showed that IPO7 overexpression inhibited PC cell apoptosis, and IPO7-siRNA#1 or IPO7-siRNA#2 could promote cell apoptosis. All of the experiments were performed in triplicate (*p < 0.05; **p < 0.01; ***p < 0.001).

**Fig. 3:**
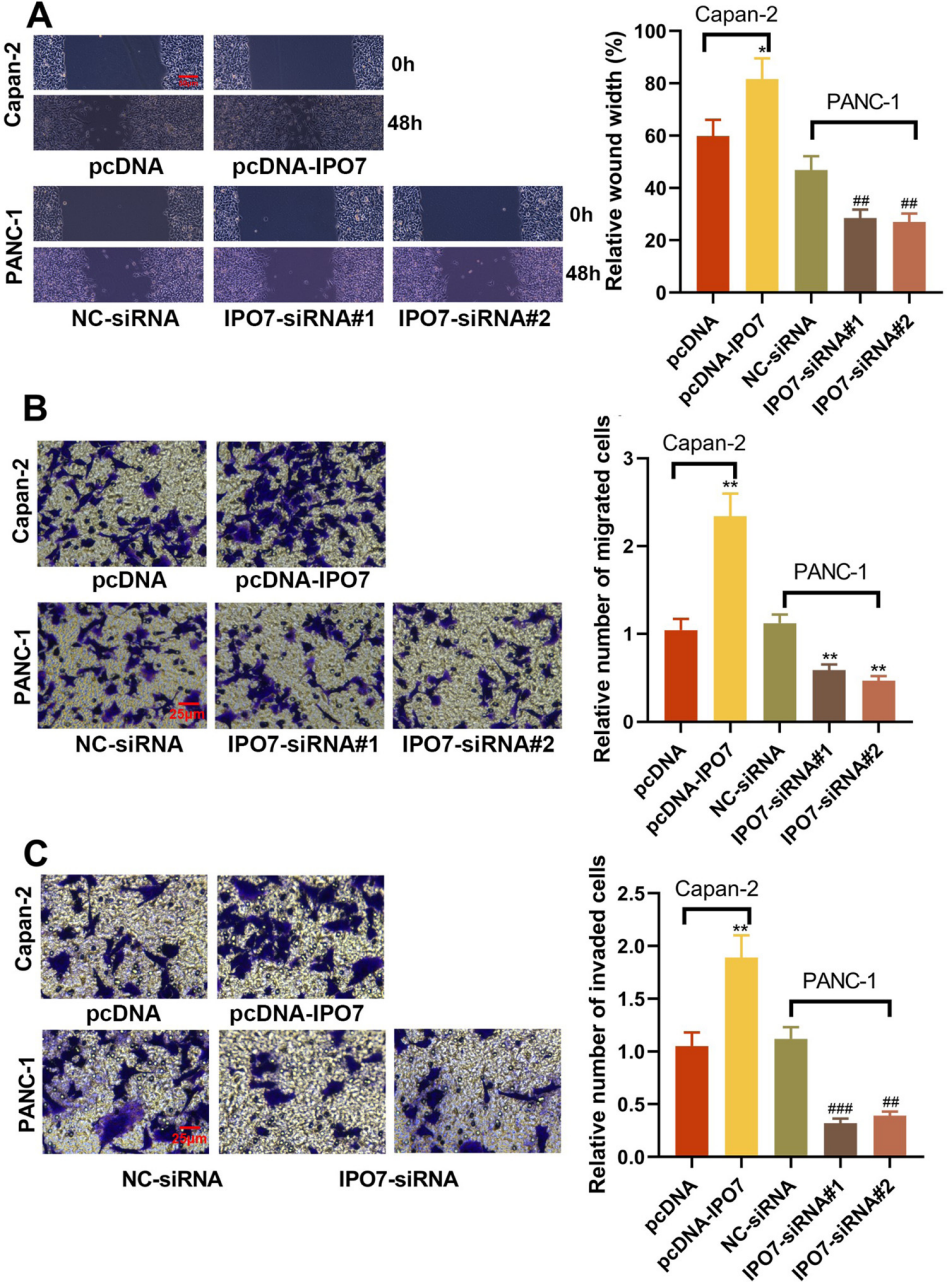
Effect of IPO7 on the migration and invasion of PC cells. (A) Wound healing experiment showed that PC cell motility was promoted in the pcDNA-IPO7 group and decreased in the IPO7-siRNA#1 and IPO7-siRNA#2 group. (B‒C) The Transwell experiment showed that the migration and invasion of PC cells was increased in the pcDNA-IPO7 group and decreased in the IPO7-siRNA#1 and IPO7-siRNA#2 group. All of the experiments were performed in triplicate (**p < 0.01).

The relative expression of IPO7 was highest in PANC-1 cells and lowest in Capan-2 cells, so these two cell lines were selected for subsequent experiments. To verify the regulatory effect of IPO7 on the proliferation and apoptotic ability of PC cells, pcDNA-NC or pcDNA-IPO7 was transfected into Capan-2 cells, and NC-siRNA, IPO7-siRNA#1, or IPO7-siRNA#2 was transfected into PANC-1 cells. Successful transfection was confirmed by qRT-PCR and Western blotting (Fig. 2A‒B). The data of CCK-8, EdU experiments, TUNEL assay, and flow cytometry showed that IPO7 overexpression remarkably enhanced the growth and suppressed the apoptosis of Canpan-2 cells compared with the controls, while knockdown of IPO7 notably repressed the growth and enhanced the apoptosis of PANC-1 cells (Fig. 2C‒F). Moreover, the data of the wound healing assay and Transwell experiment suggested that IPO7 overexpression remarkably enhanced the motility, migration and invasion of Capan-2 cells compared with the control group, while knockdown of IPO7 remarkably suppressed the motility, migration, and invasion of PANC-1 cells (Fig. 3A‒C).

### IPO7 facilitates the malignant phenotype of PC cells by positively modulating the ERBB pathway

**Fig. 4:**
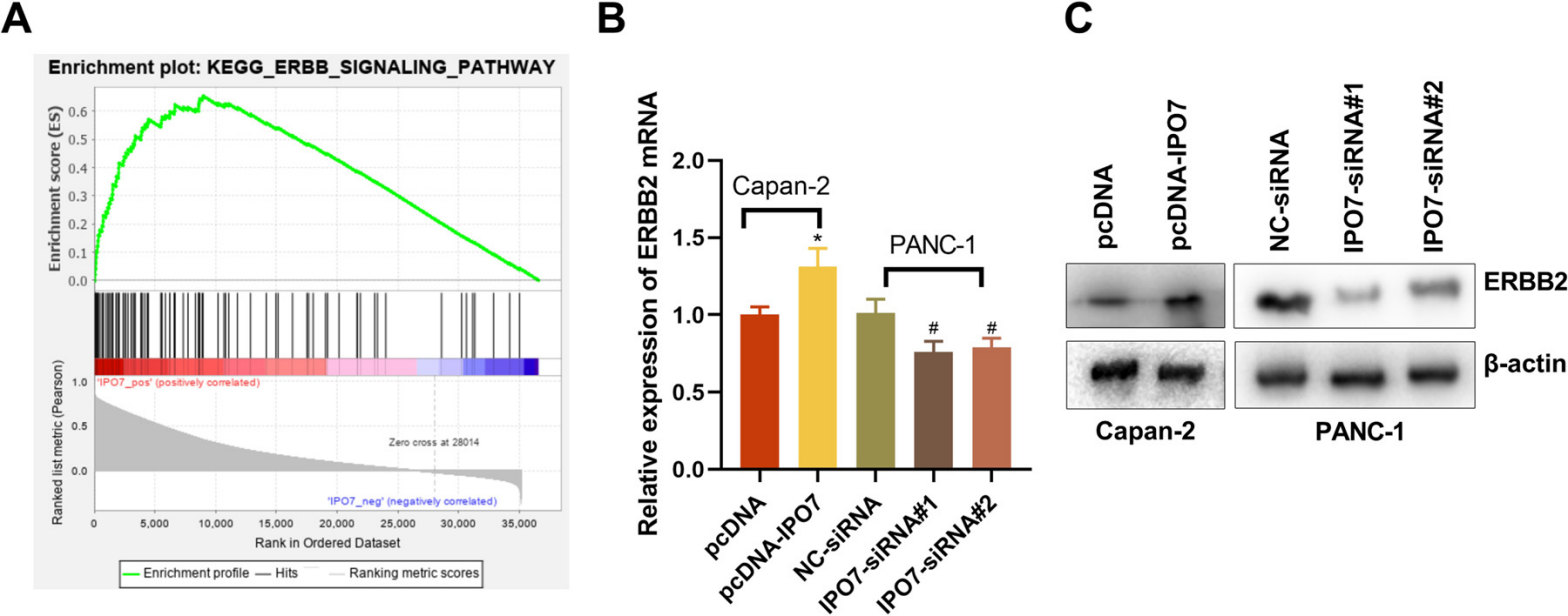
The regulatory effect of IPO7 on the ERBB pathway. (A) GSEA showed that ERBB signal pathway was related with IPO7 in PC. (B‒C) qRT-PCR and Western blotting showed that ERBB2 expression was up-regulated in pcDNA-IPO7 group and down-regulated in IPO7-siRNA#1 and IPO7-siRNA#2 group. All of the experiments were performed in triplicate (*p < 0.05).

To elucidate the downstream molecular mechanism of IPO7 in the PC development, GSEA was executed based on mRNA expression data from TCGA, and the data showed that IPO7 overexpression was positively correlated with the activation of ERBB signaling (Fig. 4A). Next, ERBB2’s expression level was also detected by qRT-PCR and Western blotting. The findings implied that ERBB2 expression was remarkably higher in pcDNA-IPO7 group and remarkably lower in IPO7-siRNA group (Fig. 4B‒C). These data implied that IPO7 could probably promote the malignant biological behaviors of PC cells through the positive modulation of ERBB pathway.

### IPO7 enhances the progression of PC in vivo

**Fig. 5:**
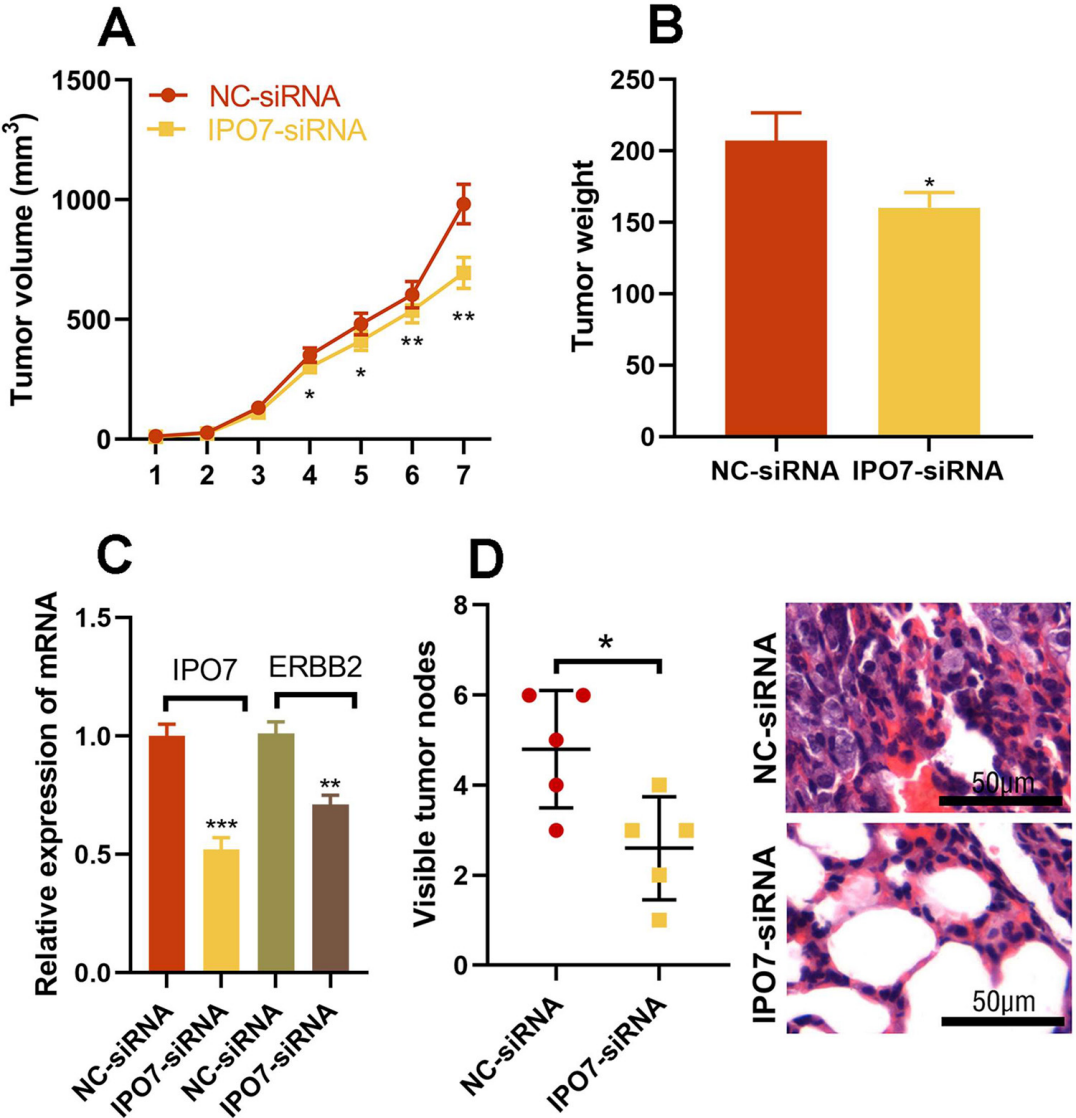
The effect of IPO7 on the development of PC *in vivo*. PANC-1 cells with IPO7 knockdown or the control PANC-1 cells were inoculated into the mice via subcutaneous injection or tail vein. (A) Tumor volumes were decreased in the IPO7-siRNA group. (B) Tumor weight were decreased in the IPO7-siRNA group. (C) qRT-PCR showed that IPO7 mRNA and ERBB mRNA expression levels were decreased in the tumor tissue of the mice in IPO7 knockdown group. (D) The lung metastasis of the mice was ameliorated in IPO7 knockdown group, and HE staining was utilized to evaluated the histopathological changes of the lung of the mice (*p < 0.05).

Next, in vivo assay was conducted to observe the biological effects of IPO7. In nude mice tumorigenicity assay, it was revealed that the tumor volume was remarkably reduced after the knockdown of IPO7 relative to the control group (Fig. 5A). Moreover, the mean weight of tumors in the IPO7-siRNA group was remarkably lower than that in the NC-siRNA group (Fig. 5B). The data of qRT-PCR showed that IPO7 and ERBB2 expression were remarkably down-modulated in the tumor tissues of IPO7 knockdown group (Fig. 5C). Furthermore, a lung metastasis model was established to evaluate the metastatic potential of PC cells *in vivo*. The data suggested that knockdown of IPO7 reduced the formation of pulmonary nodules *in vivo* (Fig. 5D), further supporting the carcinogenic role of IPO7 in PC.

## Discussion

PC is an aggressive disease with a lack of biomarkers for early diagnosis, high metastatic potential, poor chemotherapy responsiveness, and is one of the leading causes of cancer-related deaths worldwide.^16^ In recent years, more and more potential biomarkers and therapeutic targets of PC have been reported.^17^, ^18^, ^19^ In this work, IPO7 is unveiled to be remarkably up-regulated in PC. Functionally and mechanistically, IPO7 can facilitate the malignant phenotype of PC cells through ERBB pathway, suggesting a carcinogenic role for IPO7 in the development of PC.

As one of the important members of the Importin β family, IPO7 is a nuclear transport factor that can cross the nuclear envelope rapidly and bi-directionally.^20^ Additionally, IPO7 is observed to be overexpressed in diverse cancers, and it is implicated in promoting cancer progression.^21^ The protein level of IPO7 is up-modulated in primary prostate cancer cells and can participate in modulating the proliferation of cancer cells.^22^ Moreover, Forkhead box M1 (FOXM1) transcriptionally activates the expression of IPO7 to promote the nuclear import of glioma-associated oncogene homolog 1 (GLI1), and this biological process modulates the growth, migration, and invasion of glioblastoma multiforme cells *in vitro*.^23^ These studies suggest that IPO7 may act as a potential therapy target for cancer therapy. In this work, mRNA levels and protein levels of IPO7 were observed to be remarkably up-modulated in both PC tissues and cell lines. *In vitro* and *in vivo* assays showed that IPO7 could remarkably enhance the aggressiveness of PC cells. Interestingly, a recently published study also reports that IPO7 is high expression in PC, and it forms a positive feedback loop to promote PC progression.^24^ The data of this study are consistent with the present findings, suggesting IPO7 is an oncoprotein in PC.

The ERBB family, as growth factor-dependent tyrosine kinase receptors, exerts an essential effect on cell proliferation and differentiation.^25^ ERBB family members are divided into three regions: the ligand-linked region outside the cell membrane, the tyrosine kinase-activated functional region inside the membrane, and the transmembrane region.^26^, ^27^ Members of the ERBB family include ERBB1 (EFGR), ERBB2 (HER2), ERBB3 (HER3) and ERBB4 (HER4).^28^ ERBB family members have been confirmed to participate in the progression of PC. Specifically, the expression of transcription factor SRY-box transcription facto 9 (SOX9) in PC is correlated with the targets of ERBB pathway, and SOX9 can up-regulate ERBB2 expression in PC cell lines.^29^ Another study reports that neratinib exerts its tumor-suppressive effects on PC cells via reducing the expression level of ERBB1 and ERBB2.^30^ Activation of ERBB2 signaling enhances tumor growth and can act synergistically with KRAS to promote the aggressiveness of PC cells.^31^ In this work, it was revealed that IPO7 positively modulated the expression of ERBB2 in PC cells. The present data suggest that IPO7 contributes to the dysfunction of ERBB pathway in PC progression. However, the detailed mechanism by which IPO7 regulates the expression of ERBB2 requires further investigation in the following studies.

In conclusion, this work confirms that IPO7 can enhance the malignant biological behaviors of PC cells through positive regulation of the ERBB pathway and is implicated in facilitating tumor growth and lung metastasis. The present study suggests that IPO7 may be an effective biomarker and therapeutic target in the diagnosis and treatment of PC.

## Authors’ contributions

Dongqiang Xu, Ming Li: Conceived and designed the experiments.

Ming Li, Yijun Zhan: Performed the experiments.

Dongqiang Xu, Shiyun Tan: Statistical analysis.

Dongqiang Xu, Ming Li: Wrote the paper.

All authors read and approved the final manuscript.

## Ethics statement

The present study was approved by the Ethics Review Board of Renmin Hospital of Wuhan University.

## Data Availability Statement

The data used to support the findings of this study are available from the corresponding author upon request.

## Conflicts of interest

The authors declare no conflicts of interest.
